# Physiological upregulation of RAG1 expression compromises the survival of CD4^+^CD8^+^ thymocytes *in vitro*

**DOI:** 10.3389/fimmu.2026.1849780

**Published:** 2026-09-23

**Authors:** Abani Kanta Naik, Javid P. Mohammed, Rebecca A. Glynn, Craig H. Bassing, Michael S. Krangel

**Affiliations:** 1 Department of Integrative Immunobiology, Duke University School of Medicine, Durham, NC, United States; 2 Department of Pathology and Laboratory Medicine, Children’s Hospital of Pennsylvania, Perelman School of Medicine, University of Pennsylvania, Philadelphia, PA, United States

**Keywords:** cell survival, recombination activating gene 1, T cell, thymus, V(D)J recombination

## Abstract

Thymocytes upregulate expression of recombination activating genes (RAGs) upon developmental transition from the CD4^−^CD8^−^ double-negative to the CD4^+^CD8^+^ double-positive (DP) stage. Although enhanced RAG expression is necessary to generate a complete *Tcra* repertoire, here we demonstrate that elevated RAG expression simultaneously compromises DP thymocyte survival *in vitro*. Using a series of mouse models in which the expression and activity of RAG proteins varied, we attribute this apparent toxicity to elevated expression of RAG1 protein, independent of its association with RAG2 and its canonical role as a nuclease for V(D)J recombination. We propose that this toxicity accounts for the previously documented expansion of the DP compartment in mice with reduced RAG expression *in vivo*. These results indicate that DP thymocytes endure RAG1-compromised survival as a necessary trade-off for the formation of a complete *Tcra* repertoire and suggest novel aspects of RAG1 biology unrelated to V(D)J recombination.

## 1 Introduction

The recombination activating genes *Rag1* and *Rag2* encode subunits of a site-specific endonuclease complex that plays a central role in the vertebrate adaptive immune response by catalyzing V(D)J recombination. The RAG1 and RAG2 proteins assemble to form a hetero-tetramer that binds to recombination signal sequences (RSSs) adjacent to variable (V), diversity (D), and joining (J) gene segments of antigen receptor (AgR) loci and introduces double-stranded DNA breaks (DSBs) at pairs of RSSs held in a synaptic complex (1). These DSBs are then repaired by the non-homologous end joining pathway to create rearranged genes that encode the diverse arrays of antigen receptors present on T and B lymphocytes. Notably, RAG1 and RAG2 bind not only at AgR loci but also broadly across the genome (2–4). Due to the intrinsic toxicity of DSBs, mistargeting of RAG proteins creates a potential threat to lymphocyte genome integrity (3, 5).

In the RAG hetero-tetramer, RAG1 harbors the DNA binding domains and the catalytic core containing the DDE triad (D600, D708 and E962 in mouse RAG1) responsible for DNA cleavage (6). RAG2 interacts with RAG1 and with flanking DNA and, although less well-understood, is essential for DNA cleavage. Coupled RSS cleavage can be catalyzed by the minimal “core” regions of RAG1 and RAG2 (core RAG1 spanning aa 384–1008 of the 1040-aa mouse RAG1, and core RAG2 consisting of aa 1–387 of the 527-aa mouse RAG2). However, the “non-core” regions enhance the efficiency of V(D)J recombination and provide additional functions. For example, the “non-core” region of RAG2 harbors a plant homeodomain which guides RAG2 to chromatin containing H3K4me3 marks and contains a phosphorylation site that regulates cell-cycle dependent degradation (7–9). The RAG1 N-terminal “non-core” region includes sequences that mediate E3 ubiquitin ligase activity (10–13), nucleolar localization (14), and interactions with other proteins affecting RAG1 turnover (15, 16). Nevertheless, beyond the canonical RAG activities associated with AgR V(D)J recombination, the *in vivo* roles of any non-canonical RAG activities and RAG genomic and subcellular distributions are poorly understood (4, 6).

During thymocyte development, RAG expression occurs in two waves (17). RAG expression first occurs in CD4^−^CD8^−^ double-negative 2 and 3 (DN2 and DN3) thymocytes, when the gene segments at the *Tcrb*, *Tcrg* and *Tcrd* loci undergo V(D)J recombination. Following productive *Tcrb* gene rearrangement at this stage, RAG expression is downregulated in DN4 thymocytes and then upregulated as thymocytes transition to the CD4^+^CD8^+^ double-positive (DP) stage. At this phase, RAG activity is directed towards the *Tcra* locus, which undergoes multiple cycles of Vα-Jα recombination to form the *Tcra* repertoire (18). RAG expression is then permanently downregulated following positive selection (19, 20). RAG genes are similarly expressed in two waves during B cell development to catalyze V(D)J recombination of immunoglobulin heavy- and light-chain genes (21).

Cell lineage- and developmental stage-specific expression of RAG is orchestrated by distinct enhancer elements in developing T and B lymphocytes (22, 23). We and others have shown that thymocyte-specific RAG expression is driven by a cis-regulatory region called the anti-silencer (ASE) or RAG-T cell enhancer (R-Ten) (24–27). Major lymphocyte transcription factors such as E2A, Gata3, Runx1, Satb1, and Ikaros directly occupy the ASE/R-Ten and the *Rag1* and *Rag2* promoters to mediate enhancer-promoter looping and gene activation (26, 27). A striking feature of thymocyte development is that RAG genes are expressed at a substantially higher level in DP as compared to DN thymocytes. Recently, we discovered that RAG upregulation during the DN to DP transition is mediated by a DP-specific open chromatin region adjacent to the core ASE (named DPASE) and that upregulation is directed by transcription factor RORγt (28). Genetically manipulated mice lacking the DPASE expressed RAG genes in DP thymocytes but without upregulation as compared to DN thymocytes. This resulted in a reduced efficiency of *Tcra* recombination, as demonstrated by impaired usage of distal Vα and Jα gene segments. We observed a similar phenotype in mice with hemizygous expression of *Rag1* or *Rag2*, confirming that RAG expression is limiting for *Tcra* repertoire formation. Humans heterozygous for RAG mutations similarly display reduced usage of distal TRAV and TRAJ gene segments (29). Surprisingly, mice lacking the DPASE also displayed increased numbers of DP thymocytes, but this increase could not obviously be attributed to a defect in positive-selection and transition to the CD4^+^CD8^−^ or CD4^−^CD8^+^ stages (28). Thus, the mechanistic basis for this increase was unclear.

Here we report that DP thymocytes with reduced RAG expression have a survival advantage *in vitro*. Surprisingly, the toxicity resulting from elevated RAG expression was not due to mis-handling of *Tcra* locus DSBs and was wholly independent of RAG nuclease activity. Rather, we show that toxicity to DP thymocytes represents a distinct property of high level RAG1 expression that is independent of RAG2. This study suggests novel facets of RAG1 biology and indicates that DP thymocytes endure RAG1-compromised survival as a necessary trade-off for the formation of a complete *Tcra* repertoire.

## 2 Materials and methods

### 2.1 Mice

Mice lacking the DPASE (ΔDPASE) (28) and the T cell receptor alpha enhancer (ΔEα) (30) were described previously. *Rag1^−/−^* and *Rag2^−/−^* mice were obtained from The Jackson Laboratory.

Mice with a knock-in of the RAG1 D708A mutation were generated by CRISPR-Cas9-mediated gene-targeting using gRNA 5’-GGAGATGGGAGGCATCCCCAGGACTTTTAAATTCATCT-TCAGGGGCACTGGATACGATGAAAAACTTGTCCGGGAAGTAGAAGGCTTGGAAGCTTCTGGCTCAGTCTACATCTGTACACTCTGTGACACCACCCGTTTGG-3’ and ssoDNA 5’-GGAGGCCATGAAGAGCAGTGAATTAACGCTGGAGATGGGAGGCATCCCCAGGACTTTTAAATTCATCTTCAGGGGCACTGGATACGcgGAAAAACTTGTGCGAGAAGTAGAAGGCTTGGAAGCTTCTGGCTCAGTCTACATCTGTACACTCTGTGACACCACCCGTTTGGAAGCCTCTCAGAATCTTGTCTTCCACTCCA-3’ and other materials as previously described (31). Founders were backcrossed to wild-type C57BL/6 mice for two generations to ensure the propagation of a single validated mutant allele and limit potential off-target CRISPR effects. Heterozygous F2 mice were crossed to generate homozygous mice and Sanger sequencing was conducted on the genotyping PCR products to validate presence of the RAG1 D708A mutation and absence of any other mutations. Sequence-validated F2 lines were analyzed by flow cytometry using wild-type and *Rag1^−/−^* C5LBL/6 controls to confirm that *Rag1^D708A/D708A^* mice exhibited the same B and T cell developmental block as *Rag1^−/−^* mice.

All mice were housed in specific pathogen-free facilities managed by the Duke University Division of Laboratory Animal Resources or the Children’s Hospital of Philadelphia (CHOP) Department of Veterinary Resources and were used in accord with protocols approved by the Duke University and CHOP Institutional Animal Care and Use Committees.

### 2.2 Cell collection

DP thymocytes from WT, ΔDPASE, ΔEα, ΔEαΔDPASE, *Rag1^+/−^*, and *Rag2^+/−^* mice were collected by cell sorting (Astrios, Beckman Coulter) as described earlier (28). DP thymocytes from *Rag1^−/−^*, *Rag2^−/−^*, and *Rag1^D708A/D708A^* mice were obtained 10 days after a single intraperitoneal injection of 150 μg anti-CD3 antibody (145-2C11; BioLegend) into 3 week-old mice.

### 2.3 Cell survival assay

Thymocyte survival analysis was performed as described earlier (32, 33). 5x10^6^ sorted DP thymocytes were pelleted by centrifugation at 1250 RPM for 6 min at 4°C and were resuspended in 10ml of RPMI 1640 with 10% (vol/vol) FBS, 100 U/ml penicillin, 100 μg/ml streptomycin and 55 μM 2-mercaptoethanol. The cells were divided into two cell culture plates with 5 ml wells, both of which were maintained in a 37°C incubator in the presence of 5% CO_2_. One plate was used to harvest thymocytes at 24 h and the other at 48 h. Following culture, DP thymocytes were pelleted by centrifugation at 1250 RPM for 6 min at 4°C, were washed once with 1 ml of cell staining buffer (Cat. No. 420201, BioLegend) and were resuspended in 1 ml of Annexin V binding buffer (Cat. No. 422201, BioLegend). 100μl of each cell suspension was transferred to a 5 ml FACS tube to which 5μl of APC-conjugated Annexin-V (Cat. No. 640290) and 5μl of 7AAD (Cat. No. 420403) were added. Parallel 100ul cell suspensions were used for unstained, as well as Annexin V-APC and 7AAD single-stain controls. The tubes were gently mixed by tapping and were stained in the dark at 23°C. After 15 min of incubation, 400μl of Annexin V binding buffer (Cat. No. 422201, BioLegend) was added to each tube and the cell populations were analyzed by flow cytometry (Cytek Aurora) to distinguish live (Annexin V^−^ 7AAD^−^), early apoptotic (Annexin V^+^ 7AAD^−^), late apoptotic/necrotic (Annexin V^+^ 7AAD^+^) and necrotic (Annexin V^−^ 7AAD^+^) cells.

## 3 Results

### 3.1 Elevated RAG expression impairs the survival of DP thymocytes

We hypothesized that the increase in the number of DP thymocytes in DPASE-deleted mice could reflect an increase in cell survival or reduced positive selection as a consequence of impaired *Tcra* repertoire formation. To analyze differences in cell survival in the absence of positive selection, we quantified survival during *in vitro* culture of sorted WT and ΔDPASE DP thymocytes (**Figure 1A**; **Supplementary Figure 1**). After 48 h culture, ΔDPASE DP thymocytes displayed a 28% increase in survival (Annexin V^−^ 7AAD^−^) compared to WT littermate controls (**Figure 1B**).

**Figure 1:**
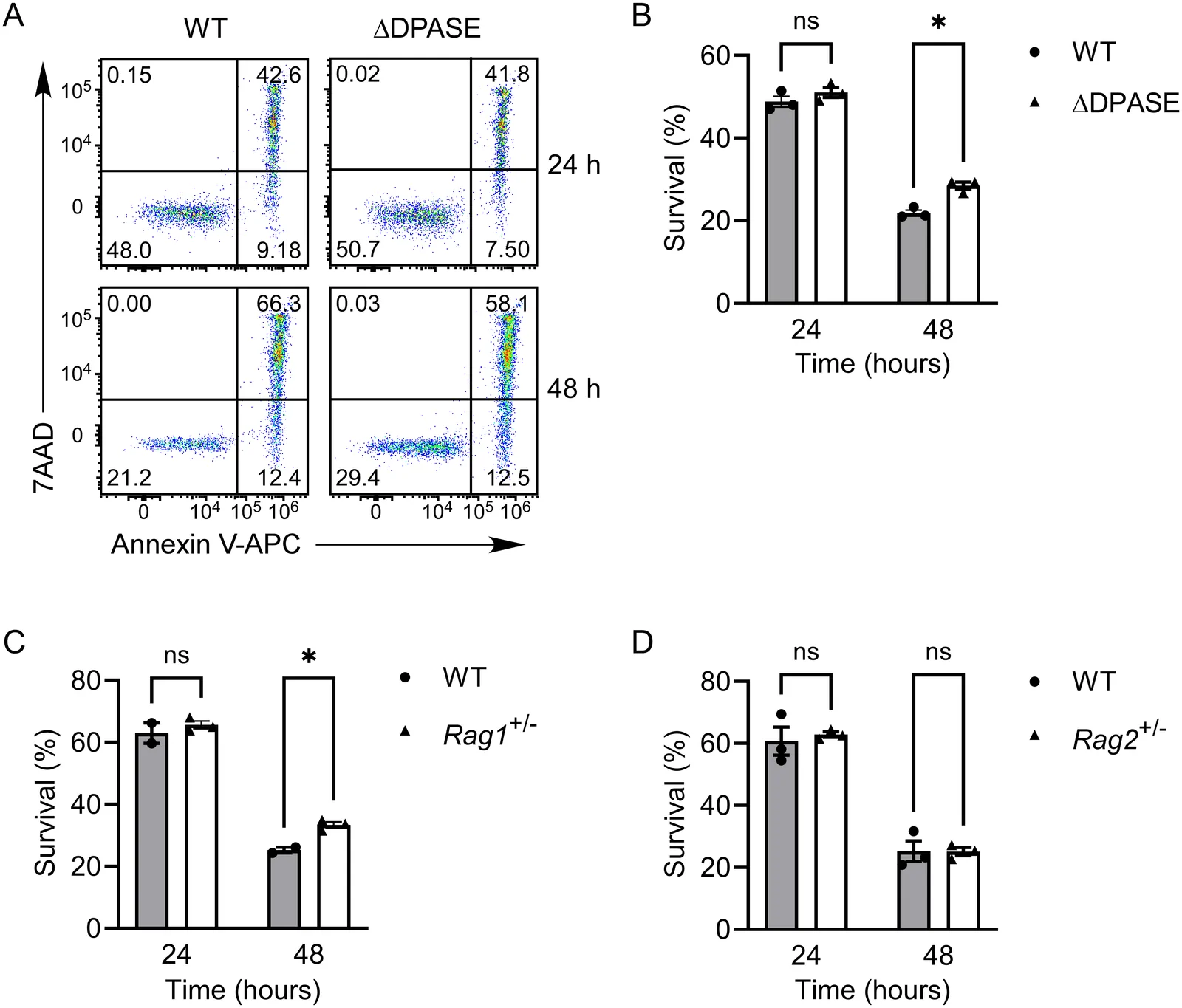
Elevated RAG expression impairs DP thymocyte survival. **(A)** Sample Annexin V and 7AAD staining profiles of DP thymocytes isolated from WT and ΔDPASE littermate mice after 24 h and 48 h of culture. Live cells (Annexin V^−^7AAD^−^) were distinguished from early apoptotic (Annexin V^+^7AAD^−^), late apoptotic/necrotic (Annexin V^+^7AAD^+^) and necrotic (Annexin V^−^7AAD^+^) cells. **(B)** Survival of WT and ΔDPASE DP thymocytes after 24 h and 48 h of culture. The data represent the mean ± SEM of three WT and three ΔDPASE littermates, compared in two independent experiments. Statistical significance was evaluated using multiple unpaired t-test. **(C)** Survival of WT and *Rag1^+/−^* DP thymocytes after 24 h and 48 h of culture. The data represent the mean ± SEM of two WT and three *Rag1^+/−^* littermates compared in two independent experiments. Statistical significance was calculated using multiple unpaired t-test. **(D)** Survival of WT and *Rag2^+/−^* DP thymocytes after 24 h and 48 h of culture. The data represent the mean ± SEM of three independent experiments comparing WT and *Rag2^+/−^* littermates. Statistical significance was evaluated using multiple unpaired t-test. ns, not significant; *P ≤ 0.05.

To further establish a causal relationship between RAG expression levels and DP thymocyte survival, we examined DP thymocytes of mice with hemizygous expression of *Rag1* or *Rag2*. Previous work had shown that thymocytes of *Rag1*^+/−^ mice display reduced expression of RAG1 protein (34). Moreover, our prior studies had strongly implied reduced expression of RAG1 and RAG2 in the respective hemizygotes, since mice hemizygous for *Rag1* or *Rag2* both displayed reductions in secondary *Tcra* recombination (28). Like ΔDPASE DP thymocytes, DP thymocytes from *Rag1*^+/−^ mice displayed significantly enhanced survival at 48 hrs of culture compared to the WT littermate controls (**Figure 1C**). However, we did not detect enhanced survival of *Rag2*^+/−^ DP thymocytes (**Figure 1D**). A potential explanation for the distinct outcomes of *Rag1*- and *Rag2*-hemizygosity is that RAG1 is much less abundant than RAG2 in thymocyte nuclei (35), and could therefore be limiting for RAG hetero-tetramer activities. However, as noted above, even mice hemizygous for *Rag2* displayed effects on secondary *Tcra* recombination (28). Alternatively, a specific pro-survival effect of *Rag1*-heterozygosity could reflect a toxic activity of RAG1 that is independent of RAG2. Regardless, these results argue strongly that physiologically elevated RAG expression compromises the survival of DP thymocytes *in vitro*.

### 3.2 RAG-dependent toxicity to DP thymocytes is independent of *Tcra* locus DSBs

The physiologic target of canonical RAG activity in DP thymocytes is the *Tcra* locus. Because the *Tcra* locus undergoes multiple rounds of biallelic Vα-to-Jα rearrangement that are facilitated by elevated RAG expression, we considered that the toxic effects of elevated RAG expression could reflect mishandling of *Tcra* locus DSBs. *Tcra* rearrangement and targeting of RAG proteins to the *Tcra* locus depend on the *Tcra* enhancer (Eα) (30, 36). Therefore, to test whether RAG cleavage activity at the *Tcra* locus was toxic for DP thymocytes, we compared survival of WT DP thymocytes with that of Eα-deleted littermates. Loss of RAG activity at the *Tcra* locus had no effect on DP thymocyte survival (**Figure 2A**). To confirm that RAG-dependent toxicity to DP thymocytes was independent of RAG activity at the *Tcra* locus, we generated mice lacking both Eα and the DPASE. In comparison to mice lacking Eα, we detected significantly improved survival of DP thymocytes isolated from littermates lacking both Eα and the DPASE (**Figure 2B**). Thus, the toxic effects of high-level RAG expression were independent of RAG activity at the *Tcra* locus.

**Figure 2:**
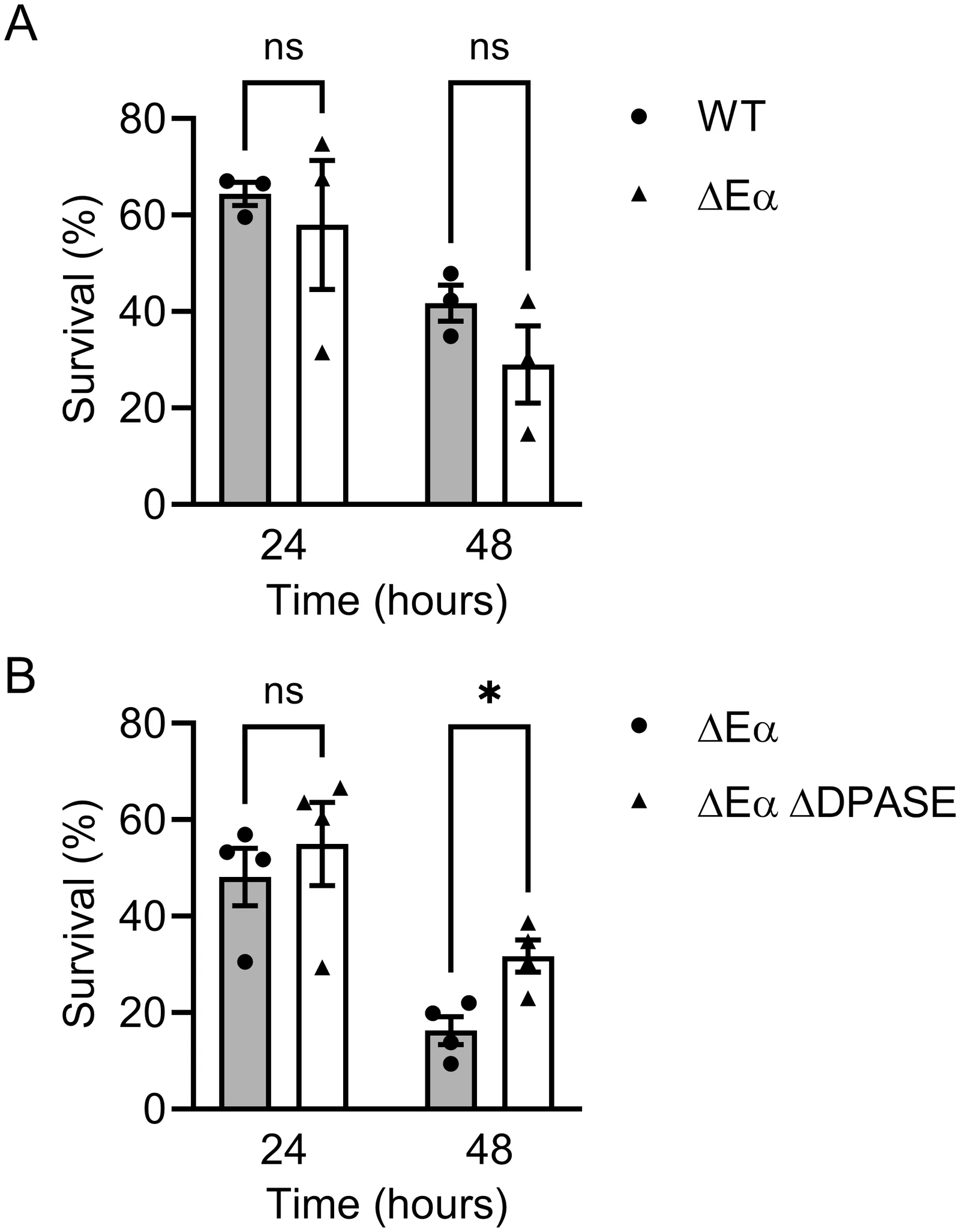
RAG-dependent toxicity to DP thymocytes is independent of *Tcra* locus DSBs. **(A)** Survival of WT and ΔEα DP thymocytes after 24 h and 48 h of culture. The data represent the mean ± SEM of three independent experiments comparing WT and ΔEα littermates. Statistical significance was evaluated by multiple unpaired t-test. **(B)** Survival of ΔEα and ΔEαΔDPASE DP thymocytes after 24 h and 48 h of culture. The data represent the mean ± SEM of 4 independent experiments comparing ΔEα and ΔEαΔDPASE littermates. Statistical significance was evaluated using multiple unpaired t-test. ns, not significant; *P ≤ 0.05.

### 3.3 RAG1 is toxic for DP thymocytes independent of its association with RAG2 and canonical RAG nuclease activity

The above experiments left open the possibilities that RAG toxicity resulted from RAG cleavage activity elsewhere in the genome (3) or from an activity of RAG1, RAG2, or the RAG hetero-tetramer that is unrelated to DSB formation. In this regard, RAG1 and RAG2 display partly distinct genomic distributions, with RAG1 targeted to chromatin decorated with H3K27Ac and RAG2 tethered to chromatin decorated with H3K4me3 (2–4). Moreover, RAG1 accumulates in the nucleolus (14). To begin to address these possibilities, we assessed the survival of *Rag1*^−/−^ and *Rag2*^−/−^ DP thymocytes. To do so, we generated compound heterozygotes that carried *Rag1^−^Rag2^+^* and *Rag1^+^Rag2^−^* alleles and then intercrossed these mice to generate *Rag1*^−/−^ and *Rag2*^−/−^ littermates. We then collected DP thymocytes for *in vitro* culture ten days after injection of anti-CD3 antibody to drive DN to DP transition. We found that DP thymocytes lacking RAG1 survived significantly better in culture than those lacking RAG2 (**Figure 3A**). These data indicate that RAG1 is toxic to DP thymocytes independent of RAG2 and independent of RAG-induced DSBs. These data align well with the distinct effects of *Rag1* and *Rag2* hemizygosity noted above (**Figures 1B, C**). We could not directly compare RAG-deficient to WT DP thymocytes because anti-CD3 injection was used to generate the former but not the latter and it was unclear how this difference might influence DP thymocyte survival *in vitro*. Anti-CD3 injection could not be used in WT mice because it induced massive depletion of the DP compartment.

**Figure 3:**
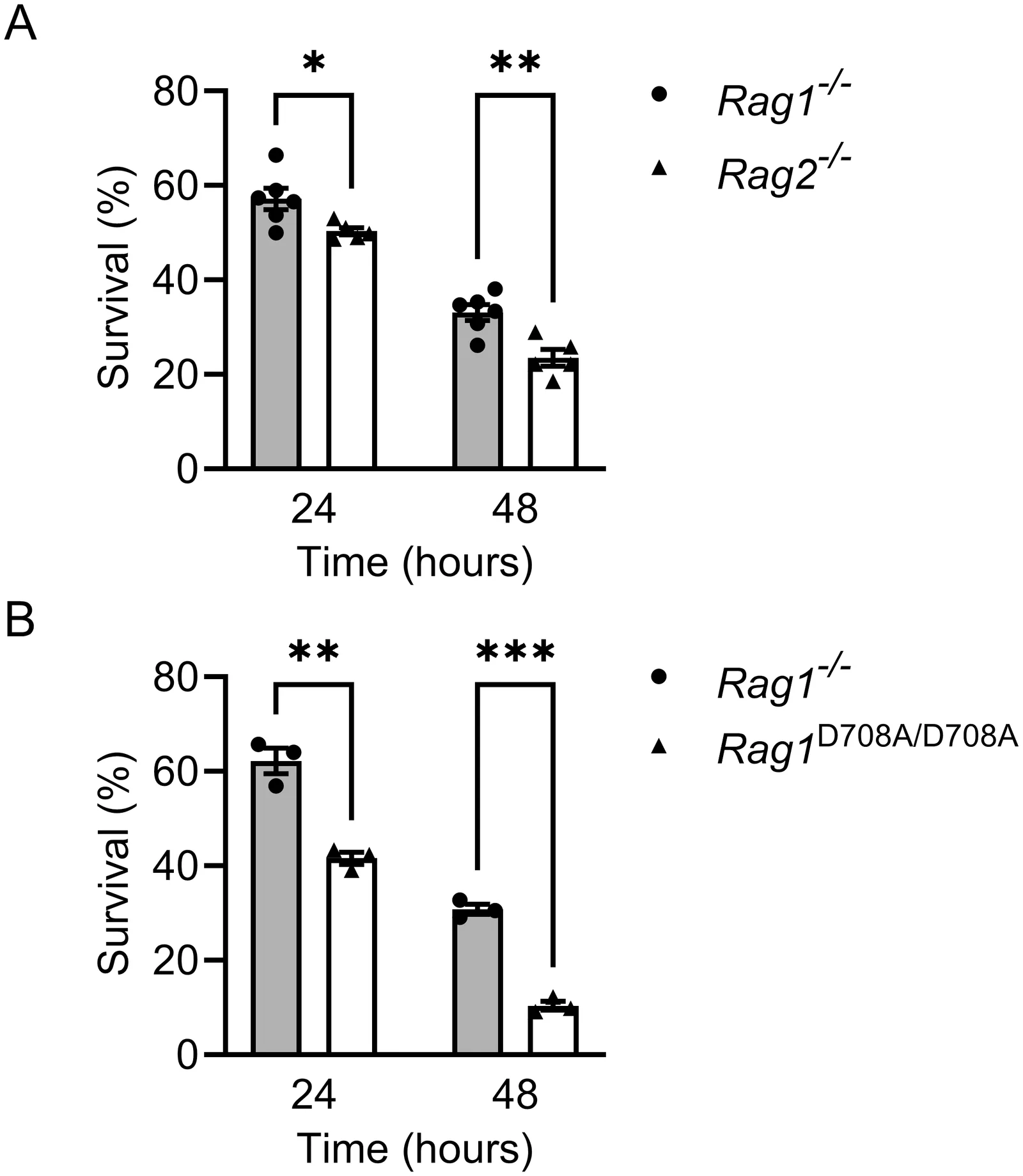
RAG1 is toxic for DP thymocytes independent of its association with RAG2 and canonical RAG nuclease activity. **(A)** DP thymocytes were obtained from *Rag1*^−/−^ and *Rag2*^−/−^ mice 10 days after anti-CD3ϵ injection and cell survival was assessed after 24 h and 48 h of culture. The data represent the mean ± SEM of 6 *Rag1*^−/−^ and 5 *Rag2*^−/−^ littermates compared in 4 independent experiments. **(B)** DP thymocytes were obtained from *Rag1*^−/−^ and *Rag1*^D708A/D708A^ mice 10 days after anti-CD3ϵ injection and cell survival was assessed after 24 h and 48 h of culture. The data represent the mean ± SEM of three independent experiments comparing *Rag1*^−/−^ and *Rag1*^D708A/D708A^ littermates. Statistical significance was evaluated using multiple unpaired t-test. *P ≤ 0.05; **P ≤ 0.01; ***P ≤ 0.001.

To provide further support for the notion that toxicity is unrelated to canonical RAG nuclease activity, we asked whether toxicity is conferred by a catalytically inactive form of RAG1. Previous studies have made use of *Rag1*^−/−^ mice carrying a BAC transgene encoding a modified form of RAG1 with mutation of critical active site residue D708 (RAG1 D708A) (2). Since physiologically normal expression was essential for our experiments, we used CRISPR/Cas9 to introduce the D708A mutation into the endogenous *Rag1* locus. We intercrossed *Rag1^D708A/−^* mice to produce *Rag1^D708A/D708A^* and *Rag1*^−/−^ littermates for survival assays. Because thymocyte development in *Rag1^D708A/D708A^* mice, as in *Rag1*^−/−^ mice, is arrested at the DN stage, we injected mice of both genotypes with anti-CD3 antibody to provoke the DN-to-DP transition. We found that sorted *Rag1^D708A/D708A^* DP thymocytes survived significantly less well in culture compared to *Rag1*^−/−^ DP thymocytes (**Figure 3B**). Thus, elevated RAG1 expression is toxic for DP thymocytes, independent of its role as a sequence-specific nuclease.

## 4 Discussion

Here, we demonstrate that the level of RAG1 expression impacts the survival of DP thymocytes *in vitro*. Improved DP thymocyte survival was demonstrated in three different genetic models of reduced RAG1 expression: *Rag1*^+/−^ mice, in which RAG1 expression should be reduced by half; ΔDPASE mice, in which RAG1 expression is reduced by about two-thirds; and *Rag1*^−/−^ mice, in which RAG1 expression is eliminated. Surprisingly, selective reductions in RAG2 expression in *Rag2*^+/−^ and *Rag2*^−/−^ mice did not similarly improve DP thymocyte survival, implying that RAG1 is toxic to DP thymocytes even in the absence of RAG2 and thus independent of hetero-tetramer formation. Consistent with this, RAG1-dependent toxicity was unrelated to hetero-tetramer-mediated nuclease activity, either at the *Tcra* locus or elsewhere in the genome, as demonstrated by continued toxicity in mice that either lack Eα or express a nuclease-dead RAG1. Together, our findings demonstrate that a nuclease-independent property or function of RAG1 impacts the survival of DP thymocytes.

Our results were remarkably consistent in revealing RAG1 dose-dependent toxicity across multiple distinct genetic comparisons. The effect of DPASE deletion was demonstrated by comparison of ΔDPASE to WT (**Figure 1B**) and ΔEαΔDPASE to ΔEα (**Figure 2B**). Similarly, the effect of reduced RAG1 expression was demonstrated by comparison of *Rag1*^+/−^ to WT (**Figure 1C**), *Rag1*^−/−^ to *Rag2*^−/−^ (**Figure 3A**), and *Rag1*^−/−^ to *Rag1^D708A/D708A^* (**Figure 3B**). Notably, models assessing reduced *Rag1* expression demonstrated survival differences that were only apparent after 48 hrs of *in vitro* culture (**Figures 1B, C**, **2B**), whereas those assessing ablated *Rag1* expression demonstrated survival differences that were of greater magnitude and were detectable at both 24 and 48 h of culture (**Figure 3A, B**). The cumulative evidence for a RAG1 dose-dependent survival effect is strong.

We believe that the toxic effects of elevated RAG1 expression detected in the *in vitro* survival assays can explain our previous observation that the DP compartment expands when RAG expression is reduced *in vivo* (28). Nevertheless, we have not directly demonstrated a survival effect *in vivo*. Moreover, our data do not allow us to draw firm conclusions regarding the mechanism of cell death in our assays, since we did not detect significant reductions in the proportion of early apoptotic (Annexin V^+^ 7AAD^−^) cells when RAG1 expression was reduced or eliminated (**Supplementary Figure 2**). That said, in the two experiments in which RAG1 expression was eliminated, there were trends towards reduced early apoptosis at 24 h that fell just short of statistical significance (*Rag1*^−/−^ vs. *Rag2*^−/−^, *P* = 0.10; *Rag1*^−/−^ vs. *Rag1*^D708A/D708A^, *P* = 0.06) (**Supplementary Figure 2**).

Our recent work revealed that upregulation of RAG expression in DP thymocytes is required to generate a complete *Tcra* repertoire, with elevated RAG expression driving the kinetics of secondary Vα-Jα rearrangements and usage of distal Vα and Jα gene segments (28). Importantly secondary *Tcra* rearrangements are limited not only by the level of RAG expression, but also by the lifespan of DP thymocytes (37). Hence, DP thymocytes are engaged in a delicate balancing act, in which the boost to *Tcra* rearrangement kinetics by elevated RAG expression is weighed against the countervailing effects of elevated RAG expression on the lifespan of DP thymocytes. We presume that DPASE-mediated RAG upregulation is tuned to hit a sweet spot that is beneficial for both *Tcra* repertoire formation and positive selection, with immediate downregulation of RAG expression upon positive selection eliminating the RAG1-mediated threat to cell survival.

Our observations leave open the question of what properties or functions of RAG1 that are independent of RAG2 and hetero-tetramer-mediated nuclease activity might impact DP thymocyte survival. Although RAG2 is present in approximately 7-fold molar excess of RAG1 in DP thymocyte nuclei, a substantial proportion of RAG1 is not associated with and does not co-localize with RAG2 (35). In this regard, the genomic distributions of RAG1 and RAG2, although overlapping, are non-identical, with RAG2 recruited to H3K4me3-decorated chromatin and RAG1 recruited to sites of H3K27ac (2–4). Moreover, RAG1 has sequences that can mediate its recruitment to, and release from the nucleolus, independent of RAG2 (14, 38). Nevertheless, to the best of our knowledge, there are no examples of RAG1-mediated effects on gene expression other than those that may occur secondary to the nuclease activity of RAG hetero-tetramers, and no clear function for nucleolar sequestration of RAG1 other than to limit V(D)J recombination (14).

The best-studied RAG2- and nuclease-independent activity of RAG1 is its intrinsic E3 ubiquitin ligase activity (10, 11). RAG1 autoubiquitination promotes RAG1 turnover (10), and mutation of either the autoubiquitination site (K233) or a site that disrupts E3 ubiquitin ligase activity (P326) results in elevated RAG1 protein in mouse thymocytes (12). If RAG1 toxicity depended on its E3 ubiquitin ligase activity, mutation of P326 might be expected to promote an increase in DP thymocyte numbers. Alternatively, if E3 ubiquitin ligase activity were unrelated to RAG1 toxicity, then elevated RAG1 expression in this mutant could cause a reduction in DP thymocyte numbers. In fact, disruption of E3 ubiquitin ligase activity caused a modest reduction in the number of DP thymocytes, but this could be explained by an apparent developmental block resulting from impaired *Tcrb* rearrangement (12, 13). Hence these experiments are ambiguous regarding the relationship between RAG1-intrinsic E3 ubiquitin ligase activity and RAG1-dependent toxicity.

RAG1 turnover is also mediated by interaction between the RAG1 N-terminal region (NTR) and VprBP, which has been shown to recruit extrinsic E3 ubiquitin ligase activity in B cells and non-lymphoid cells (15, 16, 39). Consistent with this, RAG1 lacking the NTR is highly expressed in mouse DP thymocytes. While there is little or no change in the number of DP thymocytes in these mice (12), interpretation is challenging because deletion of the NTR also results in RAG1 trapping in the nucleolus (14). Thus, any potentially toxic effects of NTR-deleted RAG1 might be neutralized by re-localization. Direct measures of DP thymocyte survival will be needed to attribute any changes in DP thymocyte number in RAG1 structural gene mutant mice to toxic effects of RAG1.

In summary, DP thymocytes tolerate toxicity mediated by elevated RAG1 gene expression as a necessary trade-off to generate a complete *Tcra* repertoire. Current information provides few clues to the mechanism of this toxicity. However, better understanding of this phenomenon may yield new insights into aspects RAG1 biology that are distinct from its canonical role in V(D)J recombination.

## Data Availability

The original contributions presented in the study are included in the article/**Supplementary Material**. Further inquiries can be directed to the corresponding author.

## References

[1] Schatz DG Swanson PC . V(D)J recombination: mechanisms of initiation. Annu Rev Genet. (2011) 45:167–202. doi: 10.1146/annurev-genet-110410-132552 21854230

[2] Ji Y Resch W Corbett E Yamane A Casellas R Schatz DG . The *in vivo* pattern of binding of RAG1 and RAG2 to antigen receptor loci. Cell. (2010) 141:419–31. doi: 10.1016/j.cell.2010.03.010 20398922 PMC2879619

[3] Teng G Maman Y Resch W Kim M Yamane A Qian J . RAG represents a widespread threat to the lymphocyte genome. Cell. (2015) 162:751–65. doi: 10.1016/j.cell.2015.07.009 26234156 PMC4537821

[4] Maman Y Teng G Seth R Kleinstein SH Schatz DG . RAG1 targeting in the genome is dominated by chromatin interactions mediated by the non-core regions of RAG1 and RAG2. Nucleic Acids Res. (2016) 44:9624–37. doi: 10.1093/nar/gkw633 27436288 PMC5175335

[5] Yu X Zhou W Chen X He S Qin M Yuan M . RAG1 and RAG2 non-core regions are implicated in leukemogenesis and off-target V(D)J recombination in BCR-ABL1-driven B-cell lineage lymphoblastic leukemia. Elife. (2024) 12:RP91030. doi: 10.7554/elife.91030.3 PMC11281782 39056282

[6] Teng G Schatz DG . Regulation and evolution of the RAG recombinase. Adv Immunol. (2015) 128:1–39. doi: 10.1016/bs.ai.2015.07.002 26477364

[7] Elkin SK Ivanov D Ewalt M Ferguson CG Hyberts SG Sun ZY . A PHD finger motif in the C terminus of RAG2 modulates recombination activity. J Biol Chem. (2005) 280:28701–10. doi: 10.1074/jbc.m504731200 15964836

[8] Matthews AG Kuo AJ Ramon-Maiques S Han S Champagne KS Ivanov D . RAG2 PHD finger couples histone H3 lysine 4 trimethylation with V(D)J recombination. Nature. (2007) 450:1106–10. doi: 10.1096/fasebj.22.1_supplement.600.2 PMC2988437 18033247

[9] Li Z Dordai DI Lee J Desiderio S . A conserved degradation signal regulates RAG-2 accumulation during cell division and links V(D)J recombination to the cell cycle. Immunity. (1996) 5:575–89. doi: 10.1016/s1074-7613(00)80272-1 8986717

[10] Jones JM Gellert M . Autoubiquitylation of the V(D)J recombinase protein RAG1. Proc Natl Acad Sci USA. (2003) 100:15446–51. doi: 10.1073/pnas.2637012100 14671314 PMC307587

[11] Yurchenko V Xue Z Sadofsky M . The RAG1 N-terminal domain is an E3 ubiquitin ligase. Genes Dev. (2003) 17:581–5. doi: 10.1101/gad.1058103 12629039 PMC196008

[12] Beilinson HA Glynn RA Yadavalli AD Xiao J Corbett E Saribasak H . The RAG1 N-terminal region regulates the efficiency and pathways of synapsis for V(D)J recombination. J Exp Med. (2021) 218:e20210250. doi: 10.21417/hab2021jem PMC8374863 34402853

[13] Burn TN Miot C Gordon SM Culberson EJ Diamond T Kreiger PA . The RAG1 ubiquitin ligase domain stimulates recombination of TCRbeta and TCRalpha genes and influences development of alphabeta T cell lineages. J Immunol. (2022) 209:938–49. doi: 10.4049/jimmunol.2001441 35948399 PMC9492648

[14] Brecht RM Liu CC Beilinson HA Khitun A Slavoff SA Schatz DG . Nucleolar localization of RAG1 modulates V(D)J recombination activity. Proc Natl Acad Sci USA. (2020) 117:4300–9. doi: 10.1073/pnas.1920021117 32047031 PMC7049140

[15] Kassmeier MD Mondal K Palmer VL Raval P Kumar S Perry GA . VprBP binds full-length RAG1 and is required for B-cell development and V(D)J recombination fidelity. EMBO J. (2012) 31:945–58. doi: 10.1038/emboj.2011.455 22157821 PMC3280554

[16] Schabla NM Perry GA Palmer VL Swanson PC . VprBP (DCAF1) regulates RAG1 expression independently of Dicer by mediating RAG1 degradation. J Immunol. (2018) 201:930–9. doi: 10.4049/jimmunol.1800054 29925675 PMC6084458

[17] Wilson A Held W MacDonald HR . Two waves of recombinase gene expression in developing thymocytes. J Exp Med. (1994) 179:1355–60. doi: 10.1084/jem.179.4.1355 8145048 PMC2191458

[18] Carico Z Krangel MS . Chromatin dynamics and the development of the TCRalpha and TCRdelta repertoires. Adv Immunol. (2015) 128:307–61. doi: 10.1016/bs.ai.2015.07.005 26477370

[19] Turka LA Schatz DG Oettinger MA Chun JJ Gorka C Lee K . Thymocyte expression of RAG-1 and RAG-2: termination by T cell receptor cross-linking. Science. (1991) 253:778–81. doi: 10.1126/science.1831564 1831564

[20] Brandle D Muller C Rulicke T Hengartner H Pircher H . Engagement of the T-cell receptor during positive selection in the thymus down-regulates RAG-1 expression. Proc Natl Acad Sci USA. (1992) 89:9529–33. doi: 10.1073/pnas.89.20.9529 PMC50165 1329099

[21] Grawunder U Leu TM Schatz DG Werner A Rolink AG Melchers F . Down-regulation of RAG1 and RAG2 gene expression in preB cells after functional immunoglobulin heavy chain rearrangement. Immunity. (1995) 3:601–8. doi: 10.1016/1074-7613(95)90131-0 7584150

[22] Kuo TC Schlissel MS . Mechanisms controlling expression of the RAG locus during lymphocyte development. Curr Opin Immunol. (2009) 21:173–8. doi: 10.1016/j.coi.2009.03.008 19359154 PMC2676217

[23] Miyazaki K Miyazaki M . The interplay between chromatin architecture and lineage-specific transcription factors and the regulation of Rag gene expression. Front Immunol. (2021) 12:659761. doi: 10.3389/fimmu.2021.659761 33796120 PMC8007930

[24] Yannoutsos N Barreto V Misulovin Z Gazumyan A Yu W Rajewsky N . A cis element in the recombination activating gene locus regulates gene expression by counteracting a distant silencer. Nat Immunol. (2004) 5:443–50. doi: 10.1038/ni1053 15021880

[25] Hao B Naik AK Watanabe A Tanaka H Chen L Richards HW . An anti-silencer- and SATB1-dependent chromatin hub regulates Rag1 and Rag2 gene expression during thymocyte development. J Exp Med. (2015) 212:809–24. doi: 10.1084/jem.20142207 PMC4419350 25847946

[26] Naik AK Byrd AT Lucander ACK Krangel MS . Hierarchical assembly and disassembly of a transcriptionally active RAG locus in CD4(+)CD8(+) thymocytes. J Exp Med. (2019) 216:231–43. doi: 10.1084/jem.20181402 30545902 PMC6314527

[27] Miyazaki K Watanabe H Yoshikawa G Chen K Hidaka R Aitani Y . The transcription factor E2A activates multiple enhancers that drive Rag expression in developing T and B cells. Sci Immunol. (2020) 5:eabb1455. doi: 10.1126/sciimmunol.abb1455 32887843

[28] Naik AK Dauphars DJ Corbett E Simpson L Schatz DG Krangel MS . RORgammat up-regulates RAG gene expression in DP thymocytes to expand the Tcra repertoire. Sci Immunol. (2024) 9:eadh5318. doi: 10.1126/sciimmunol.adh5318 38489350 PMC11005092

[29] Bosticardo M Dobbs K Delmonte OM Martins AJ Pala F Kawai T . Multiomics dissection of human RAG deficiency reveals distinctive patterns of immune dysregulation but a common inflammatory signature. Sci Immunol. (2025) 10:eadq1697. doi: 10.1126/sciimmunol.adq1697 39792639 PMC12087669

[30] Sleckman BP Bardon CG Ferrini R Davidson L Alt FW . Function of the TCR alpha enhancer in alphabeta and gammadelta T cells. Immunity. (1997) 7:505–15. doi: 10.1016/s1074-7613(00)80372-6 9354471

[31] Wu GS Yang-Iott KS Klink MA Hayer KE Lee KD Bassing CH . Poor quality Vbeta recombination signal sequences stochastically enforce TCRbeta allelic exclusion. J Exp Med. (2020) 217:e20200412. doi: 10.1084/jem.20200412 32526772 PMC7478721

[32] Chen L Foreman DP Sant'Angelo DB Krangel MS . Yin Yang 1 promotes thymocyte survival by downregulating p53. J Immunol. (2016) 196:2572–82. doi: 10.4049/jimmunol.1501916 26843327 PMC4779672

[33] Sun Z Unutmaz D Zou YR Sunshine MJ Pierani A Brenner-Morton S . Requirement for RORgamma in thymocyte survival and lymphoid organ development. Science. (2000) 288:2369–73. doi: 10.1126/science.288.5475.2369 10875923

[34] Xu M Ito-Kureha T Kang HS Chernev A Raj T Hoefig KP . The thymocyte-specific RNA-binding protein Arpp21 provides TCR repertoire diversity by binding to the 3'-UTR and promoting Rag1 mRNA expression. Nat Commun. (2024) 15:2194. doi: 10.1038/s41467-024-46371-z 38467629 PMC10928157

[35] Zhang YH Shetty K Surleac MD Petrescu AJ Schatz DG . Mapping and quantitation of the interaction between the recombination activating gene proteins RAG1 and RAG2. J Biol Chem. (2015) 290:11802–17. doi: 10.1074/jbc.m115.638627 25745109 PMC4424321

[36] Ji Y Little AJ Banerjee JK Hao B Oltz EM Krangel MS . Promoters, enhancers, and transcription target RAG1 binding during V(D)J recombination. J Exp Med. (2010) 207:2809–16. doi: 10.1084/jem.20101136 21115692 PMC3005232

[37] Guo J Hawwari A Li H Sun Z Mahanta SK Littman DR . Regulation of the TCRalpha repertoire by the survival window of CD4(+)CD8(+) thymocytes. Nat Immunol. (2002) 3:469–76. doi: 10.1038/ni791 11967541

[38] Spanopoulou E Cortes P Shih C Huang CM Silver DP Svec P . Localization, interaction, and RNA binding properties of the V(D)J recombination-activating proteins RAG1 and RAG2. Immunity. (1995) 3:715–26. doi: 10.1016/1074-7613(95)90061-6 8777717

[39] Schabla NM Swanson PC . The CRL4VPRBP(DCAF1) E3 ubiquitin ligase directs constitutive RAG1 degradation in a non-lymphoid cell line. PLoS One. (2021) 16:e0258683. doi: 10.1371/journal.pone.0258683 34648572 PMC8516306

