# Supplementary material for: Physiological upregulation of RAG1 expression compromises the survival of CD4+CD8+ thymocytes in vitro
Source: Front Immunol. 2026 Sep 23;17:1849780. doi: 10.3389/fimmu.2026.1849780 (PMC13642656; doi:10.3389/fimmu.2026.1849780)
Supplement: Supplementary file 1 [file DataSheet1.pdf]

Supplementary Material

Physiologic upregulation of RAG1 expression compromises the survival of $CD4^+CD8^+$ thymocytes *in vitro*

Abani Kanta Naik, Javid P. Mohammed, Rebecca A. Glynn, Craig H. Bassing, Michael S. Krangel

1 Supplementary Figures

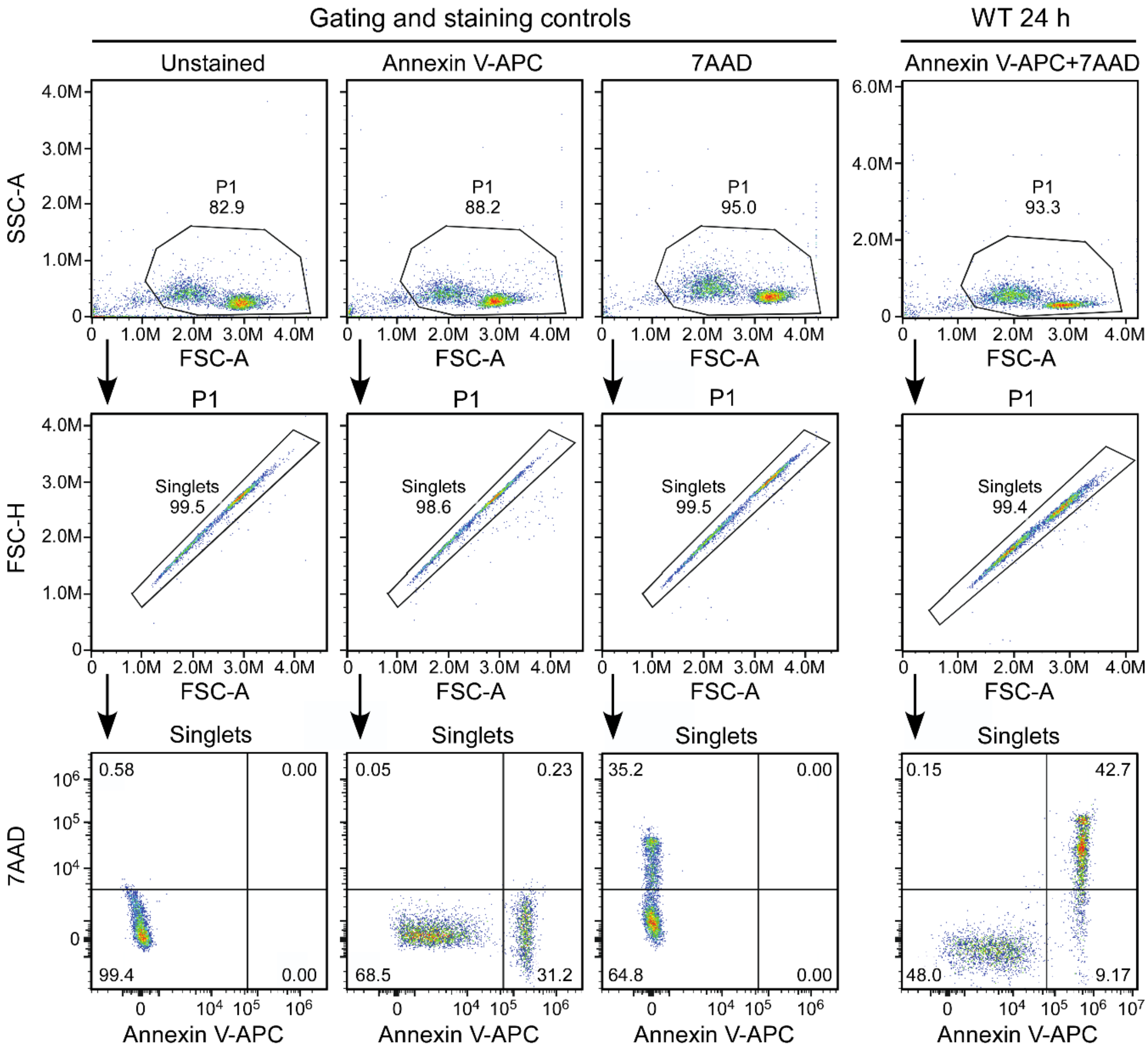

Supplementary Figure 1. Gating scheme for flow cytometric analysis of $CD4^+CD8^+$ thymocyte survival in culture. Debris and doublets were excluded by analysis of SSC-A versus FSC-A and FSC-H versus FSC-A. Left three columns, unstained, Annexin V-APC, and 7AAD single stain controls used to set gates. Right column, gating scheme for analysis of double-stained WT 24 h sample shown in Figure 1A.

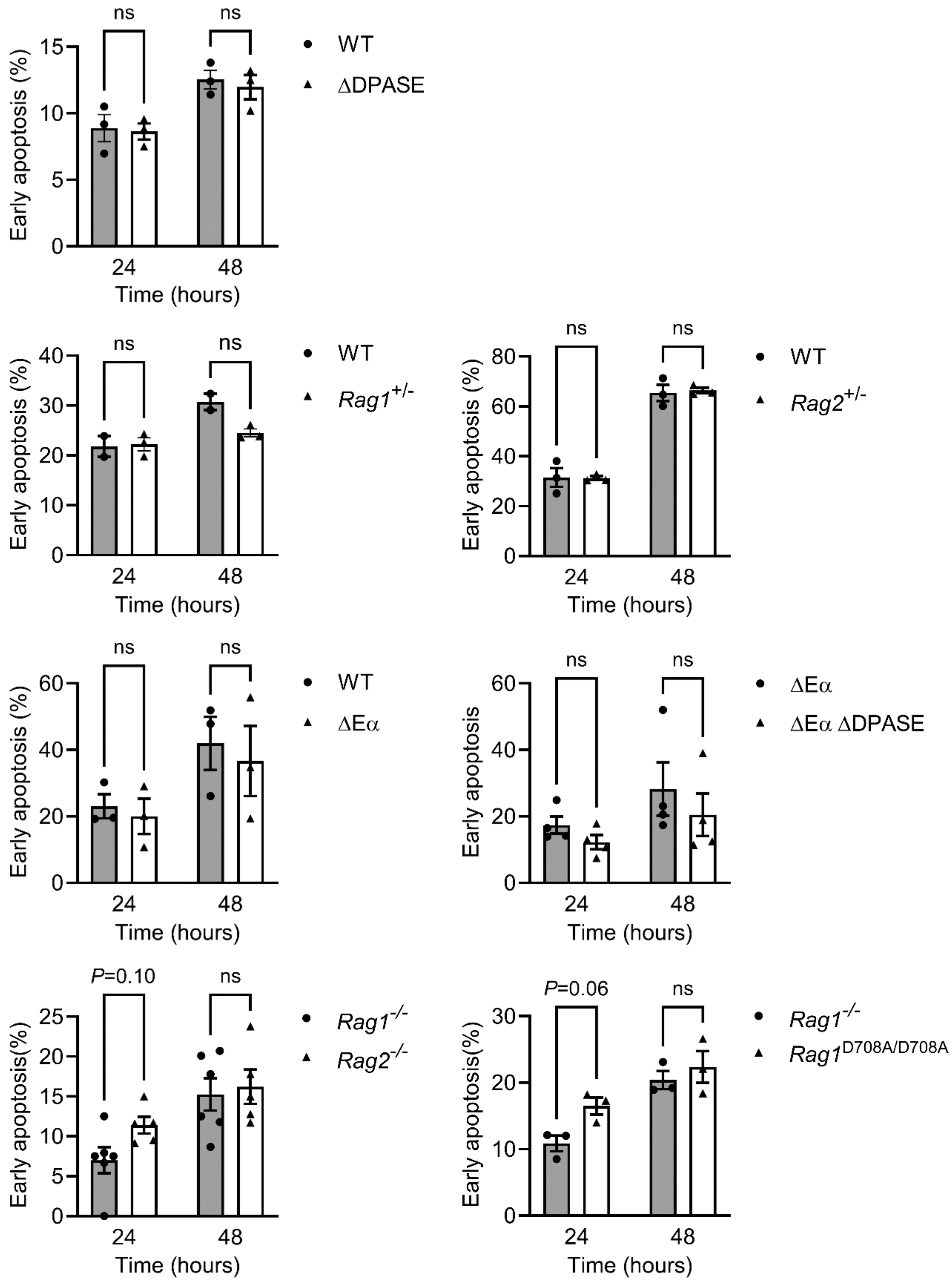

Supplementary Figure 2. Quantification of early apoptosis (Annexin V$^+$ 7AAD$^-$) in all genetic comparisons. Note trends towards reduced early apoptosis at 24 h in *Rag1*$^{-/-}$ DP thymocytes that fell short of statistical significance (bottom row).
